# Multiple Aspects of ATP-Dependent Nucleosome Translocation by RSC and Mi-2 Are Directed by the Underlying DNA Sequence

**DOI:** 10.1371/journal.pone.0006345

**Published:** 2009-07-23

**Authors:** Joke J. F. A. van Vugt, Martijn de Jager, Magdalena Murawska, Alexander Brehm, John van Noort, Colin Logie

**Affiliations:** 1 Department of Molecular Biology, NCMLS, Radboud University, Nijmegen, The Netherlands; 2 Physics of Life Processes, Leiden Institute of Physics, Leiden University, Leiden, The Netherlands; 3 Institut für Molekularbiologie und Tumorforschung, University of Marburg, Marburg, Germany; Oregon State University, United States of America

## Abstract

**Background:**

Chromosome structure, DNA metabolic processes and cell type identity can all be affected by changing the positions of nucleosomes along chromosomal DNA, a reaction that is catalysed by SNF2-type ATP-driven chromatin remodelers. Recently it was suggested that *in vivo*, more than 50% of the nucleosome positions can be predicted simply by DNA sequence, especially within promoter regions. This seemingly contrasts with remodeler induced nucleosome mobility. The ability of remodeling enzymes to mobilise nucleosomes over short DNA distances is well documented. However, the nucleosome translocation processivity along DNA remains elusive. Furthermore, it is unknown what determines the initial direction of movement and how new nucleosome positions are adopted.

**Methodology/Principal Findings:**

We have used AFM imaging and high resolution PAGE of mononucleosomes on 600 and 2500 bp DNA molecules to analyze ATP-dependent nucleosome repositioning by native and recombinant SNF2-type enzymes. We report that the underlying DNA sequence can control the initial direction of translocation, translocation distance, as well as the new positions adopted by nucleosomes upon enzymatic mobilization. Within a strong nucleosomal positioning sequence both recombinant *Drosophila* Mi-2 (CHD-type) and native RSC from yeast (SWI/SNF-type) repositioned the nucleosome at 10 bp intervals, which are intrinsic to the positioning sequence. Furthermore, RSC-catalyzed nucleosome translocation was noticeably more efficient when beyond the influence of this sequence. Interestingly, under limiting ATP conditions RSC preferred to position the nucleosome with 20 bp intervals within the positioning sequence, suggesting that native RSC preferentially translocates nucleosomes with 15 to 25 bp DNA steps.

**Conclusions/Significance:**

Nucleosome repositioning thus appears to be influenced by both remodeler intrinsic and DNA sequence specific properties that interplay to define ATPase-catalyzed repositioning. Here we propose a successive three-step framework consisting of initiation, translocation and release steps to describe SNF2-type enzyme mediated nucleosome translocation along DNA. This conceptual framework helps resolve the apparent paradox between the high abundance of ATP-dependent remodelers per nucleus and the relative success of sequence-based predictions of nucleosome positioning *in vivo*.

## Introduction

Nucleosomal DNA is strongly bound to the histone octamer and is in this way occluded from most DNA binding proteins, including RNA and DNA polymerase machineries. Hyperacetylation of histone tails can by itself decrease nucleosomal DNA occlusion by about seven fold [1]. A second mechanism to increase nucleosomal DNA accessibility involves mechanical remodeling and is performed by evolutionary conserved SNF2 enzymes that belong to the SFII superfamily of nucleic acid-stimulated ATPases [2]. The SNF2 ATPases can be classified according to their protein domains. SWI/SNF-type complexes, such as the yeast RSC complex, harbour bromodomains that can bind acetylated lysines [3]–[5]. They have been implicated in transcription initiation [6], as well as elongation [7], [8] and permit cellular identity determination, including mammalian embryonic stem cell identity maintenance and differentiation [9], [10]. SWI/SNF subtype complexes have also been implicated in chromosome transmission [11]–[14] and DNA repair [15]–[20], underlining their roles as tumour suppressors and implying that catalyzed exposure and subsequent restoration of nucleosomal DNA by SWI/SNF complexes are central to many eukaryotic chromosome based processes [21]. On the other hand, CHD-type SNF2 remodelers, like Mi-2, bear chromodomains that can bind methylated histone lysines [22], [23], although this has not been demonstrated for *Drosophila's* Mi-2 chromodomains, which have been postulated to bind DNA [24]. Mi-2 can associate with histone deacetylase activity and plays important roles in transcriptional repression during cell growth and differentiation [25]. Mi-2 is targeted to chromatin via protein-protein interactions. These include Mi-2 binding to methylated DNA binding domain (MBD) containing proteins, directly contacting sequence specific transcriptional repressors and interacting with the SUMO moiety of SUMOylated transcription factors [26]–[30].

Although different SNF2 remodeler sub-types are involved in diverse chromosomal processes, they share the ability to remodel nucleosomal chromatin [31]. ATP hydrolysis cycles by nucleosome remodelers are in the 10 to 100 milliseconds range and can be induced by DNA, histones, or combinations thereof, depending on the ATPase and associated subunits [32]. To date, biochemical and structural insights have converged on a universal model on the mechanism of nucleosome repositioning [33]–[36]. First, the remodeler binds to two or more locations on the (extra-)nucleosomal DNA and histone octamer, after which the ATPase translocates linker DNA into the nucleosome, by forming a small DNA loop or by inducing DNA twist or a combination thereof, which then propagates over the surface of the histone octamer resulting in the repositioning of the nucleosome [37]. We refer to nucleosome repositioning as the displacement of the histone octamer relative to the DNA. The exact binding locations of remodelers, whether DNA loops or twist are involved, whether the remodeler pushes or pulls the histone octamer and what parameters determine the new position of the nucleosome are aspects of this model that are still under debate and may vary among the different SNF2 remodeler sub-types.

To what extent the remodeling mechanism and resulting nucleosome repositioning are influenced by the underlying DNA sequence is also not clearly resolved. Recent work has provided compelling evidence to suggest that DNA sequence can dictate the new positions adopted by histone octamers upon enzymatic remodeling [38]–[41]. However, among remodelers striking differences have been observed concerning their preference of nucleosome repositioning on short stretches of DNA containing nucleosome positioning elements. In some cases the nucleosome was repositioned to the DNA end (recombinant ISWI) or over the DNA end (RSC, SWI/SNF); other remodelers preferred a more central position (CHD1, Mi-2, multi-subunit ISWI) [38], [39], [41]–[44]. To what extent DNA ends and/or DNA sequence *per se* influenced the catalyzed nucleosome repositioning was not fully resolved by these studies.

In the present study, a better understanding of the mechanism and kinetics of nucleosome repositioning was obtained by a single nucleosome assembled on the 601 nucleosome positioning sequence [45] flanked by relatively long DNA arms. In this way, nucleosome translocation away from its start position was not influenced by the immediate presence of DNA ends, which gave us the opportunity to separate DNA end effects from DNA sequence effects. Furthermore, the issue of remodeler specificity of nucleosome repositioning was addressed by comparing two different SNF2-type enzymes; native RSC complex from *S. cerevisiae* and recombinant Mi-2 ATPase from *D. melanogaster*. We employed single molecule imaging by AFM as well as high resolution native poly-acrylamide gel electrophoresis (PAGE) to resolve and quantify the mechanism and kinetics of catalyzed nucleosome translocation. Both SNF2-type remodelers are strongly influenced by the 601 sequence with respect to initial translocation direction, processivity and final octamer positioning. Unlike recombinant Mi-2, native RSC efficiently translocated histone octamers beyond the influence of the 601 sequence, at the same time repositioning nucleosomes over a larger distance when beyond the influence of this sequence. Overall, the outcome of remodeling appears to be strongly influenced by underlying DNA sequence, revealing an interplay between remodeler intrinsic and DNA specific properties to control ATPase induced nucleosome dynamics.

## Results

### RSC-DNA complexes conceal large stretches of DNA

**Figure 1:**
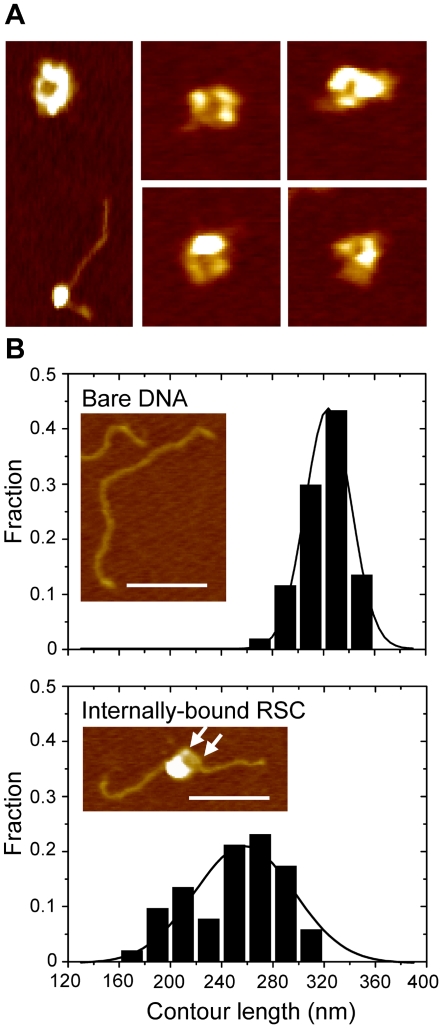
RSC structure and DNA interaction. A) AFM images of typical RSC complexes and one next to a nucleosome. Image height is 100 nm, z range is 4 nm. B) Histograms of the contour lengths of 1 kbp bare DNA (top, *N* = 106) and 1 kbp DNA with RSC bound internally (bottom, *N* = 52) with corresponding AFM images. Scale bar of AFM images is 100 nm, z-range 4 nm. White arrows indicate the two lobes of RSC that align along DNA.

Before describing the kinetics of RSC-nucleosome remodeling, we first characterized the structures of RSC, RSC-DNA and RSC-nucleosome complexes to assess the reaction stoichiometry and to get a broad overview of the reaction mechanism. These complexes were visualized with tapping mode Atomic Force Microscopy (AFM). We incubated 1 nM RSC with 21 nM 1 kbp linear DNA without ATP. Individual RSC complexes could clearly be resolved, containing a central cavity, surrounded by at least three globular subunits (Figure 1A). This central cavity appears large enough to accommodate a nucleosome, as shown in Figure 1A and as recently shown by others [35], [36]. The average volume of the RSC complexes attached to either the surface or the DNA measured 1449±325 nm^3^ (*N* = 20), in excellent agreement with the expected 1450 nm^3^ of a single complex, based on the molecular weight of this 17-subunit complex of 1.197 MDa (www.yeastgenome.org) and an average protein volume of 0.73 cm^3^/g [46]. Though the dimensions of nanometer sized features in AFM images are always affected by tip-convolution [47] and adhesion artefacts [48] it would seem that these effects cancel in this case. RSC was not homogeneously distributed over the DNA. In 48 out of 100 RSC-DNA complexes only one extruding DNA end could be resolved, suggesting a substantial affinity for DNA ends. This result could be reproduced both with and without glutaraldehyde fixation, showing that fixation has no influence on the observed end preference of RSC binding. However, we cannot exclude the enrichment of RSC-DNA end complexes by deposition biases. The large size of the RSC-DNA complex did not allow verification by independent experimental approaches. Such a bias was investigated and excluded though for *Eco*RI-DNA complexes [49]. Note that ATP was lacking in these experiments, ruling out active DNA translocation by RSC towards the ends.

The geometry of DNA-bound RSC was often the same, with two lobes of RSC aligned on the DNA (Figure 1B, Figure S1). This suggests these lobes are involved in DNA binding. The DNA footprint of RSC was further analysed by comparing the contour length of DNA-RSC complexes with that of bare DNA (Figure 1B). The average contour length of 1 kbp DNA measured 322±17 nm (*N* = 106), which is in good agreement with the expected length of 0.34 nm per bp. Upon binding of RSC to a central position on the DNA, the contour length reduced to 251±35 nm (*N* = 52). Because the trajectory of the DNA in the RSC complex is obscured, we assumed the shortest distance between the entry and the exit point of the complex. The contour length of end-bound DNA-RSC complexes was 269±51 nm (*N* = 48), also substantially smaller than that of bare DNA (Figure S1). The DNA length that RSC conceals, *i.e.* 53–71 nm or 156–209 bp, roughly matches half the circumference of RSC, which has a diameter of approximately 40 nm. Together with the absence of free DNA loops this suggests that DNA is strongly bent and wrapped in or around the RSC complex.

### AFM characterization of RSC nucleosome complexes in the presence and absence of ATP

**Figure 2:**
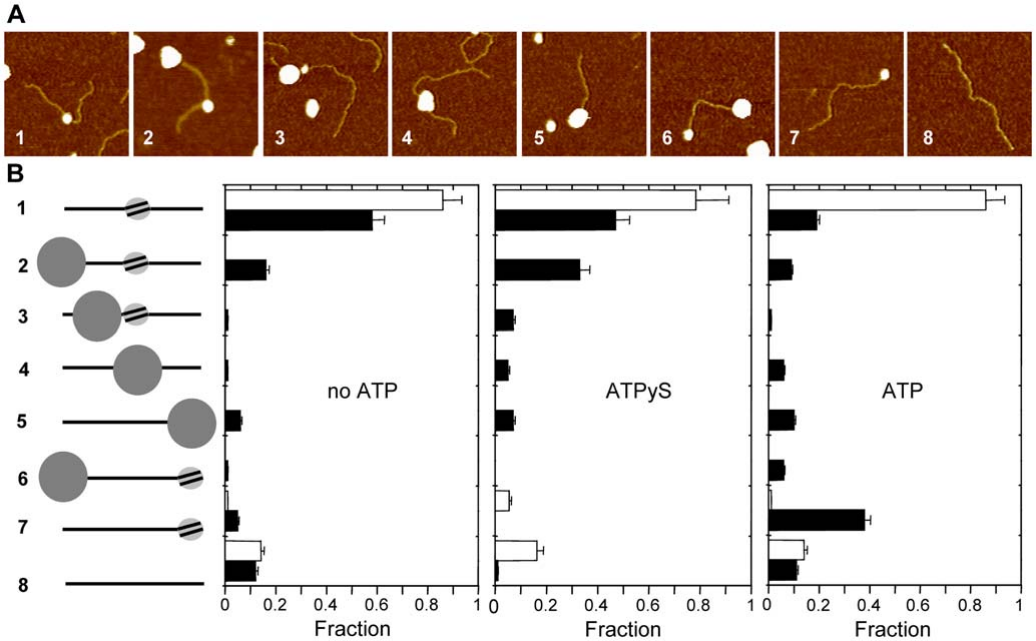
Structural analysis of RSC-mononucleosome interaction. RSC was offered a centrally positioned nucleosome with 240 bp arms (ratio RSC: mononucleosome 1∶3) for ½ hour±4 mM ATP(γS). A) AFM images of the 8 designated categories of RSC-DNA-nucleosome interactions, *i.e.* 1) nucleosome internally bound; 2) nucleosome internally bound and RSC end bound; 3) RSC and nucleosome internally and separately bound, 4) RSC internally bound; 5) RSC end bound; 6) RSC and nucleosome bound to opposite DNA ends; 7) end bound nucleosomes; 8) bare DNA. Images are 200×200 nm, z-range 4 nm. B) Quantification of the 8 RSC-substrate categories when remodeling without ATP, with 4 mM ATPγS, and with 4 mM ATP. The eight categories are expressed as the fraction of total analyzed molecules. White bars indicate result without RSC and ATP. Black bars indicate result with RSC±ATP(γS). No ATP: *N* = 133 for white bars, *N* = 139 for black bars. ATPγS: *N* = 36 for white bars, *N* = 73 for black bars. ATP: *N* = 133 for white bars, *N* = 289 for black bars.

We studied the interaction of RSC with nucleosomes using a single nucleosome reconstituted on a 601 nucleosomal positioning element flanked by two relatively long DNA arms of 236 and 252 bp. Typically 85–90% of the mononucleosomal substrate contained a nucleosome at the centrally positioned 601 sequence, 10–15% was bare DNA, and in some reconstitutions <5% featured a nucleosome at one of the DNA ends. Bearing in mind the RSC footprint on bare DNA, these long DNA arms allowed us to discriminate to what part of the nucleosomal substrate RSC is bound. We characterized the RSC-nucleosome interaction by designating eight substrate-complex categories and compared these categories in the presence or absence of ATP or ATPγS, a non-hydrolysable analogue of ATP (Figure 2A). The presence of a nucleosome inside the RSC cavity could not be resolved, preventing unambiguous structural interpretation of the complexes in categories 4 and 5. Consistent with the large RSC footprint on bare DNA, the DNA contour length in some RSC nucleosome complexes, especially category 5, was visibly shorter than that of the bare substrate.

In the absence of ATP, 72% of the RSC- bound complexes had RSC attached to DNA, and RSC could be clearly distinguished from the nucleosome (Figure 2B, ‘no ATP’, categories 2, 3 and 6). In the presence of ATPγS the majority (76%) of the RSC-substrate complexes was also distinctively bound to DNA (Figure 2B, ‘ATPγS’, categories 2, 3 and 6). Although categories 2, 3 and 6 make up 18% and 40% of all detected molecules, respectively, this result suggests that on these substrates it is bare DNA, not the nucleosome that is the preferential binding site for RSC. Furthermore, as with the RSC bare DNA complexes discussed above, most of the mononucleosomal substrates had RSC positioned at one of the DNA ends (categories 2, 5 and 6).

Efficient repositioning of nucleosomes by RSC was dependent on ATP hydrolysis, as observed by a large increase in end-positioned nucleosomes at the expense of centrally positioned nucleosomes (Figure 2B, compare categories 1–3 with 6 and 7). RSC did not appear to remove histone octamers from the DNA, because the amount of bare DNA did not increase after addition of RSC with or without ATPγS or ATP (Figure 2B, category 8). Moreover, without ATP 25% of the substrate was bound to RSC (Figure 2B, ‘no ATP’, categories 2 to 6). By adding ATPγS the fraction of RSC-bound substrate doubled to 52%, whereas with ATP 32% of the substrate was RSC-bound (Figure 2B, ‘ATPγS’ and ‘ATP’, categories 2 to 6), suggesting ATPγS induces a more stable RSC substrate interaction than with or without ATP. Furthermore, we observed an increase in nucleosome-bound RSC complexes upon addition of ATP, from 28% without ATP and 23% with ATPγS to 50% with ATP (Figure 2B, categories 4 and 5), suggesting that ATP hydrolysis enhances RSC-nucleosome interaction.

### RSC pushes nucleosomes over hundreds of base pairs

**Figure 3:**
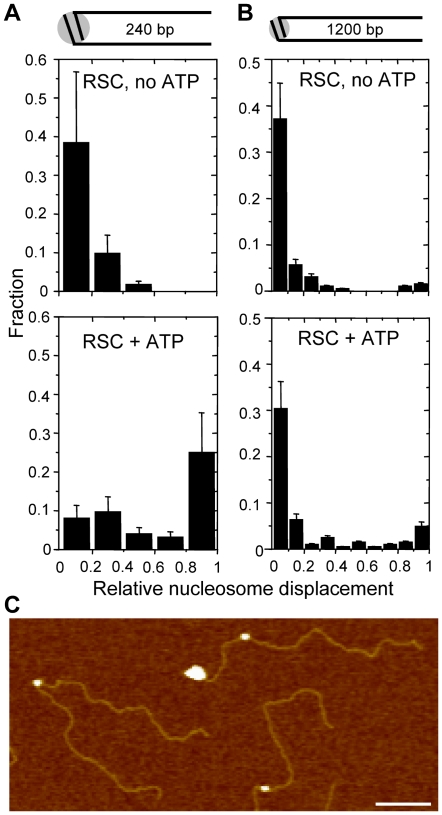
Quantification of remodeled nucleosome position. The nucleosome position was determined relative to the normalized DNA contour length. The histograms show the relative nucleosome displacement when remodeling without (top) or with (bottom) ATP. We could not discriminate to which DNA arm each nucleosome was translocated. The relative nucleosome displacement is 0 for the DNA center from where the nucleosome translocation is initiated and 1 for the DNA end. A) Mononucleosome with 240 bp arms with (*N* = 124) or without (*N* = 112) ATP. Bin size 48 bp. B) Mononucleosome with 1200 bp arms with (*N* = 204) or without (*N* = 194) ATP. Bin size 120 bp. C) AFM image of mononucleosomes with 1200 bp arms of which one with RSC bound to a DNA end. Scale bare is 100 nm, z-range 4 nm.

The remodeling potency of RSC is further demonstrated by its ability to reposition a single nucleosome over at least 1200 bp (Figure 3). The accumulation of nucleosomes at the DNA end indicates that RSC does not move nucleosomes away from the DNA end. Careful tracing of the DNA contour revealed that DNA in remodeled nucleosomal templates appeared on average 13 nm, or 38 bp, longer than that in centrally positioned nucleosomes (197±26 nm (*N* = 49) vs. 184±26 nm (*N* = 62), Figure S2). We observed a 5% decrease in nucleosome volume (420±81 nm^3^ vs 402±94 nm^3^). This indicates that no histone proteins were lost as H2A-H2B dimer dissociation results in a much larger volume loss [50], [51]. It has been reported before that RSC, like SWI/SNF, positions nucleosomes slightly over the DNA end [38], [43], [52], [53]. The fact that RSC is apparently not able to translocate the nucleosomes from the DNA ends suggests that RSC needs free DNA upstream of the nucleosome translocation direction and thus ‘pushes’ nucleosomes rather than ‘pulling’ them.

Notably, in the several hundreds of RSC-DNA and RSC-nucleosome complexes that we imaged we observed no DNA looping, in contrast to the results of others [54] and contrary to what we measured using a type I restriction enzyme [55]. It is only when the ratio RSC:DNA molecules was increased significantly that we detected DNA loops that protruded from aggregates of multiple RSC complexes (data not shown). The shape and dimensions of the RSC-DNA complexes that we visualized here, together with pronounced remodeling activity, indicate therefore that the formation of large DNA loops is not required for nucleosome remodeling by single RSC complexes. Rather, our results are consistent with the hypothesis that DNA segments, in the order of perhaps several tens of bp long are being translocated over the surface of RSC-bound nucleosomes.

### RSC repositions nucleosomes in multiple turnovers

Quantifying the position of the nucleosomes before and after 30 min of remodeling by RSC revealed the progression of nucleosome translocation along the DNA arms (Figure 3). To avoid inaccurate assessment of the nucleosome position only the substrates without RSC were considered. Figure 3A shows the relative nucleosome position along 240 bp arms. In the absence of ATP all nucleosomes were centrally located. The relatively large width of the central position distribution should be attributed to the limited accuracy of the tracing, which is in the same order as the bin size of 48 bp. In the presence of ATP about 50% of the nucleosomes were translocated to the DNA ends (Figure 3A). Importantly, approximately 10% of the nucleosomes was found in between the start position and DNA end, demonstrating that RSC does not necessarily slide the nucleosomes in one run to the DNA end but instead may dissociate from the nucleosome and rebind in multiple turnovers before reaching an end. Apparently, the ratio between the translocation velocity and the dissociation rate, *i.e.* the nucleosome translocation processivity, is thus smaller than the 240 bp length of the DNA arms.

### Nucleosomes are moderately trapped in the 601 positioning element

The limited nucleosome translocation processivity that we observed on the substrate with 240 bp arms (240/240 bp substrate) predicts that translocation efficiency over longer distances should decrease dramatically. Not only is the fraction of nucleosomes that are shifted to the DNA end in one run expected to decrease exponentially with arm length, in each subsequent interaction with RSC half of the nucleosomes would be shifted back in the reverse direction, rendering overall translocation reactions over long distances extremely inefficient. To test this hypothesis, a single nucleosome with five times longer DNA arms was reconstituted and tested for remodeling efficiency under identical reaction conditions (Figure 3B and C). Although the fraction of nucleosomes trapped on a DNA end decreased from 50% on the 240/240 bp substrate to 10% on the 1200/1200 bp substrate, this decrease is fivefold less than expected based on the processivity on the 240 bp arms. Importantly, a small but significant fraction of 0.06% of the nucleosomes (*N* = 6 vs. *N* = 0 for substrates prior to remodeling) were found between the nucleosome start position and a DNA end. Nevertheless, the majority of nucleosomes located on the 1200/1200 bp mononcleosomes remained at or in the vicinity of the initial nucleosome position. Taken together, these results suggest that the processivity of nucleosome translocation is higher outside the 601 element than within it.

**Figure 4:**
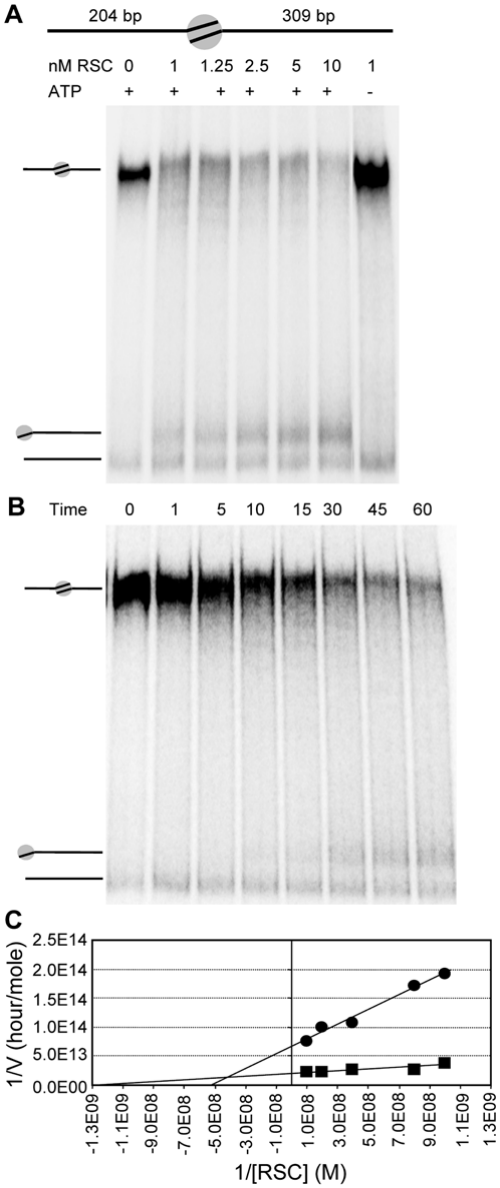
RSC titration and time course on centrally positioned nucleosomes. A) Native 4% acrylamide gel with 0–10 nM RSC titration on 7.7 nM nucleosomes with a 204 and a 309 bp arm for 1 hour with or without 1 mM ATP. B) Native 4% acrylamide gel with 1 mM ATP and 5 nM RSC on 7.7 nM nucleosomes with a 204 and 309 bp arm at time points indicated in minutes. C) Lineweaver-Burk plot from the RSC titration.

To observe the RSC induced nucleosome repositioning with a higher resolution and better statistics than with AFM, we performed time course and enzyme titration experiments using native PAGE. With this technique the band shift relative to naked DNA increases with the distance of the nucleosome from the DNA ends (Figure 4). Nucleosomes were reconstituted on radioactively end-labelled DNA templates harbouring a 601 element with DNA arms of 205 and 309 bp. Consistent with our AFM results, reconstitution yielded between 85–90% of the DNA template with a nucleosome at the 601 sequence and on average 10% bare DNA. Titration of RSC resulted in an increased amount of end positioned nucleosomes with increasing RSC concentration (Figure 4A). The fraction of bare DNA remained constant, reproducing the data obtained by AFM and showing that histone octamers were not displaced from the DNA under these reaction conditions. Time course experiments revealed that detectable levels of end-nucleosomes were obtained after 10 minutes using 5 nM RSC (Figure 4B), and after 30 minutes when 1 nM RSC was used (data not shown). Because the initial nucleosome was positioned slightly off centre, we could also resolve how fast nucleosomes were mobilized from the starting positing, resulting in bands above and below the starting position (Figure 4A and B). Consistent with the AFM data, only little radioactive signal was detected between the bands corresponding to the starting and the end positioned nucleosome at any RSC concentration or time point. Together, these results indicate that nucleosomes are moderately trapped in the 601 element, resulting in a larger band width surrounding the initial nucleosome, but are efficiently translocated over larger distances once they escape this region.

In keeping with previous affinity measurements for RSC and SWI/SNF [56]–[58], classical kinetic analysis of the RSC titration using Michaelis-Menten kinetics (Figure 4C) revealed K_M_ values of 0.5 and 2 nM and V_max_ values of 15 to 50 femtomoles per hour, for the disappearance of the initial nucleosome and the increase of the end nucleosome, respectively (Figure 4C). These observations reinforce the notion that the nucleosome processivity along the length of DNA templates we studied here is not constant.

### Nucleosomes are repositioned with 10 bp intervals within the 601 positioning element

**Figure 5:**
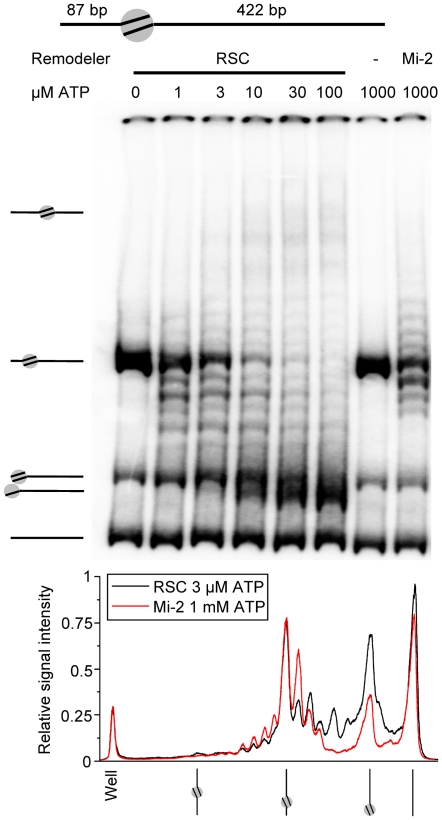
Stepwise nucleosome remodeling on off-centre nucleosomes. Native 4% acrylamide gel with 5 nM RSC or 10 nM Mi-2 on 11.5 nM nucleosomes with a 87 and a 422 bp arm for 1 hour and a 0–100 µM ATP titration (RSC) or 1 mM ATP (Mi-2) (Upper panel). The graph in the lower panel shows the relative signal intensity of two acrylamide gel lanes, one with RSC remodeled mononucleosomes (3 µM ATP) and one with Mi-2 remodeled mononucleosomes (1 mM ATP).

Careful inspection of the gels in Figure 4 revealed a subtle band pattern around the 601 positioning sequence that was not resolved by AFM. In the absence of ATP this band pattern was not observed, confirming that they are products of ATP-dependent remodeling (Figure 4A). However, the band distribution around the slightly off-centred nucleosome was too compressed to unambiguously quantify individual bands. Furthermore, its quasi-central position did not allow us to determine the direction of nucleosome translocation. We therefore produced a nucleosome with one short (87 bp) and one long (422 bp) DNA arm. Using this substrate a clear ladder of nucleosome positions could be resolved after remodeling (Figure 5). One of the bands that appeared after RSC remodeling, ran faster through the gel than the end-positioned nucleosomes that were a side product in this reconstitution, confirming that RSC pushes nucleosomes over the DNA end (Figure 5). Titration of ATP had no effect on the position of the bands, it only changed the number of bands of the nucleosome ladder, demonstrating ATP concentration independence of the catalyzed nucleosome ‘step size’.

Is this observed band pattern characteristic for RSC or is it a general feature of ATP-dependent remodelers? To answer this question we compared the reaction products of RSC to those of *Drosophila* Mi-2. Figure 5 shows that RSC and Mi-2 induce seven identical preferred nucleosome positions; three on the short DNA arm and four on the long DNA arm. We therefore conclude that the obtained nucleosome positioning pattern is neither remodeler specific nor ATP concentration-dependent.

**Figure 6:**
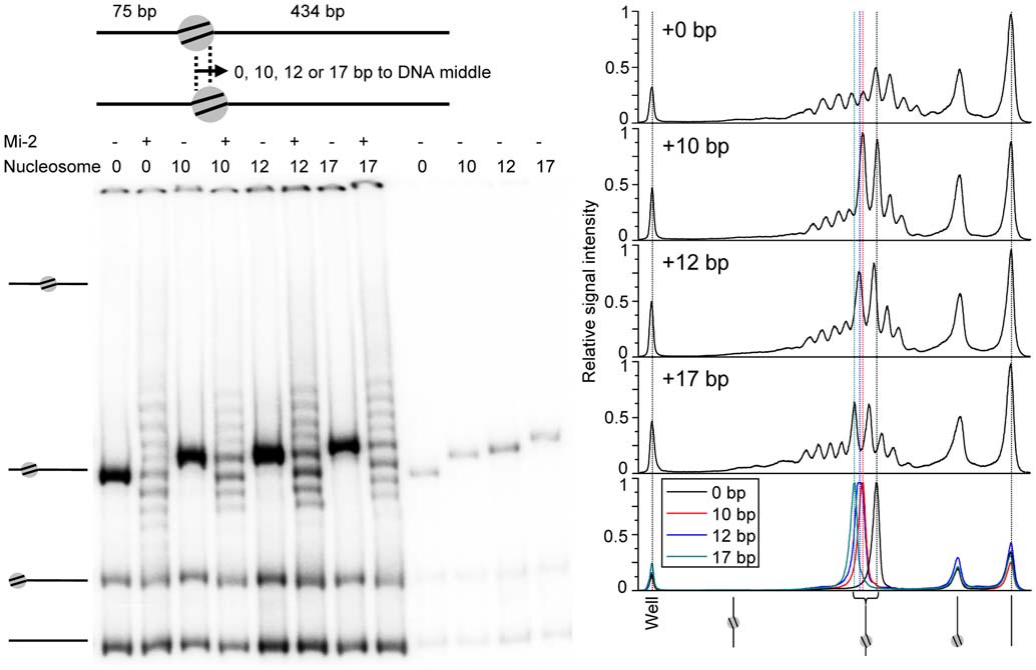
Step size of nucleosome repositioning within the 601 sequence. Native 4% acrylamide gel with 10 nM Mi-2 on 11.5 nM mononucleosomes with a 75 and a 434 bp arm for 1 hour and 1 mM ATP (left panel). The graphs in the right panel show the relative signal intensity of the acrylamide gel lanes remodeled with Mi-2 (upper 4 graphs) and the not remodeled lanes (lower graph), whereby a relative signal intensity of 1 corresponds to the highest peak in the graph. All lanes in all graphs have been aligned using the well peak and bare DNA peak as references. The dotted lines indicate the peaks of the well, the bare DNA and the 0 bp, 10 bp, 12, bp and 17 bp mononucleosomes.

To quantify the periodicity of nucleosome remodeling we reconstituted nucleosomes that were positioned 0, 10, 12 and 17 bp towards the DNA centre, and subjected these to remodeling by Mi-2. Figure 6 demonstrates that the periodicity of the remodeling induced nucleosome ladder is 10/11 bp.

Surprisingly, it appeared that under limiting ATP conditions (Figure 5) RSC had a pronounced preference to reposition nucleosomes by 20 bp rather than 10 bp relative to the nucleosome's initial position. This implies that RSC favours catalyzing single 15–25 bp nucleosomal over approximately 10 bp steps.

**Figure 7:**
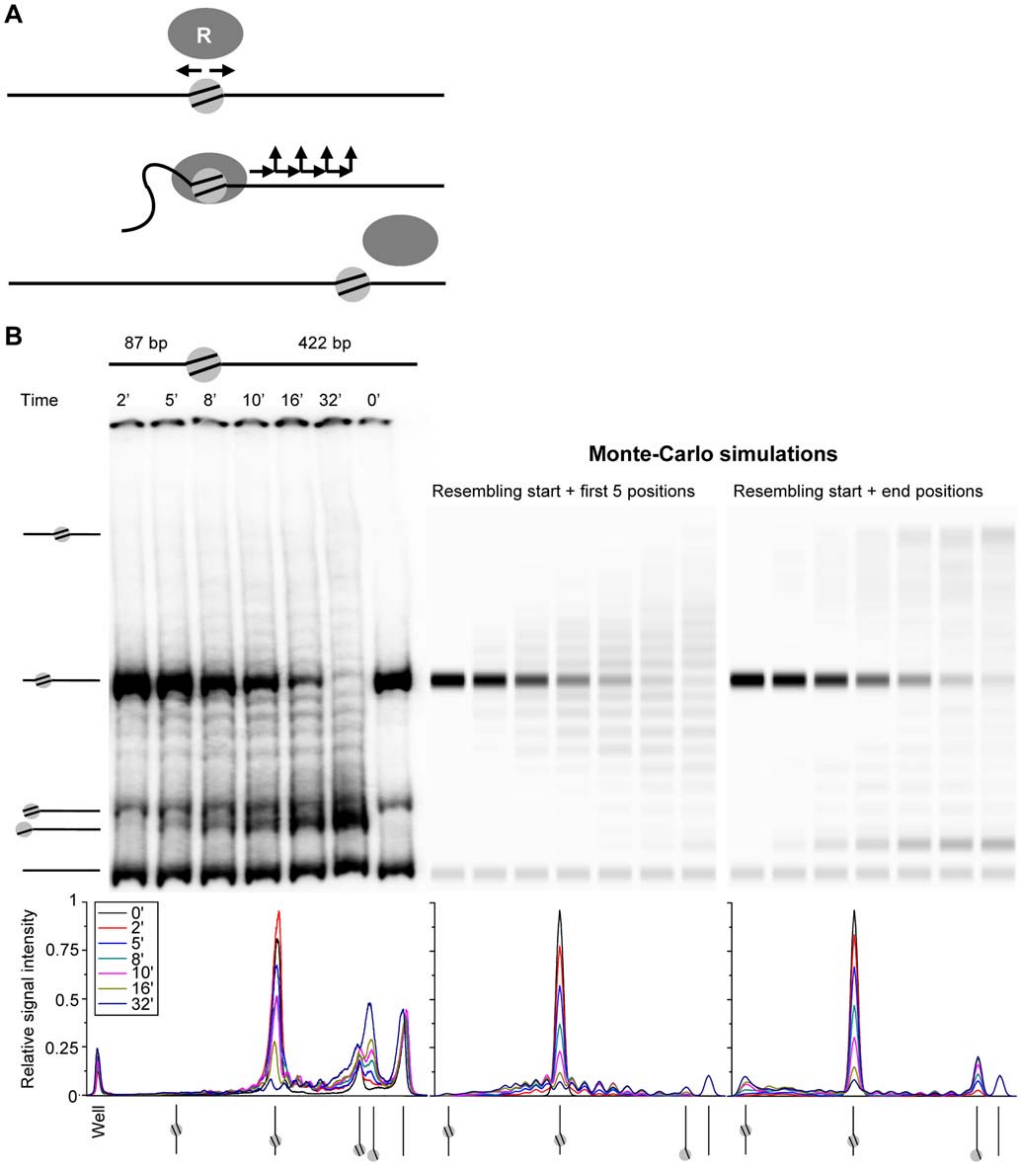
Markov chain model of nucleosome translocation. A) Markov chain model showing remodelers (R) upon binding can translocate nucleosomes in either direction along DNA with a fixed step size and Poisson distributed step number before releasing from the substrate. B) Native 4% acrylamide gel of a time course of 5 nM RSC on 11.5 nM mononucleosomes with a 87 and a 422 bp arm and 1 mM ATP and Monte-Carlo simulations assuming RSC is not able to reposition the nucleosome from DNA ends, that it initially binds the nucleosome and translocates it with 10 bp steps with a processivity of two (middle panel) or eight (right panel) steps. The lower panel reveals graphs of the relative signal intensity of the acrylamide gel (lower left panel) and the Monte-Carlo simulations (lower middle and right panels), whereby a relative signal intensity of 1 corresponds to the highest peak in the graph.

Finally, one conspicuous difference between Mi-2 and RSC is that native RSC complex appeared to be more processive than recombinant Mi-2 since RSC moved nucleosomes further away from the nucleosome's starting position than Mi-2, even when Mi-2 was supplied with sufficient ATP (Figure 5 and compare Figure 7B and S3).

### Monte Carlo simulations substantiate limited nucleosome processivity in the 601 sequence

To obtain more insight on the nucleosome processivity of remodelers, we used a Monte Carlo simulation to reproduce nucleosome translocation by RSC and Mi-2 and thereby capture the essential ingredients of remodeling kinetics. We modelled catalyzed nucleosome remodeling with a Markov chain of events as sketched in Figure 7A. The Markov chain closely follows the sequence of structures deduced from AFM images captured during remodeling, shown in Figure 2. In this model, upon binding to a nucleosomal substrate the remodeling enzyme has an equal probability to translocates along the DNA in either direction. When bound, enzymes progress in a single direction with a fixed step size of 10 bp until they release from the substrate. After each step the remodeler has a finite probability, as defined by the processivity, to continue with a next step or to dissociate from the substrate. The processivity is defined as the ratio between the forward and off rate. In our model the translocation distance follows a Poisson distribution, consistent with a dissociation probability that is independent of the number of translocation steps taken before.

The binding affinity, step size, forward rate and dissociation rate of remodelers are likely different on DNA and on a nucleosome. In the original model these parameters were implemented separately. We also included the binding preference of RSC for bare DNA, as observed with AFM. However, good agreement with experimental data was only obtained when the processivity of RSC on bare DNA was either very low or exceeded the length of the DNA arms, making the outcome independent of the initial remodeler binding position. Therefore, we simplified our model by assuming that the remodeler binds directly to the nucleosome. From there, it translocates the nucleosome in either direction with 10 bp intervals. Nucleosomes were fixed once positioned at a DNA end in the case of RSC while they were reflected from the DNA ends for Mi-2. The results of the simulations are displayed in a format resembling a PAGE experiment as well as band density scans, allowing direct visual comparison with our experimental data (Figure 7B, Figure S3).

Given the above constraints the only remaining free parameters in our Monte Carlo simulation are the processivity of nucleosome translocation and the number of turnovers per substrate. For Mi-2 the best agreement with experimental data was obtained with a nucleosomal processivity of one 10 bp step and a Mi-2 reaction rate of 1 turnover per nucleosome per 3 minutes (Figure S3). The good resemblance with our experimental data demonstrates that our model can indeed capture the most important features of the reaction mechanism and suggests that recombinant Mi-2 releases from the substrate after each 10 bp step.

The PAGE results obtained with RSC could not be reproduced using a single value for the processivity. We therefore reproduced the kinetics of the nucleosome within the positioning sequence separate from the kinetics of the nucleosome outside the 601 sequence. We obtained the closest resemblance to the decline of the initial nucleosome and the nucleosome ladder pattern with a processivity of two 10 bp steps per nucleosome and one turnover every 12 minutes (Figure 7B). However, with a 20 bp nucleosomal processivity we were not able to mimic the kinetics of the accumulation of end nucleosomes. It therefore seems that a processivity of 20 bp only applies to the region surrounding the nucleosomal starting position. A processivity of 80 bp per nucleosome and one turnover every 12 minutes resembled the increase in end nucleosomes for both the central and off centre nucleosome substrates, but now the characteristic 601 nucleosome band pattern was absent (Figure 7B, compare Figure 4A and S4). Taken together the simulations imply that within the influence of the 601 sequence RSC has a lower nucleosomal translocation processivity than outside the influence of this sequence, where it is more than 80 bp per remodeling event. Thus, Monte Carlo simulations confirm that for an accurate description of the processivity of nucleosome repositioning it is imperative to take the underlying DNA sequence into account.

### DNA sequence determines the nucleosome translocation direction

**Figure 8:**
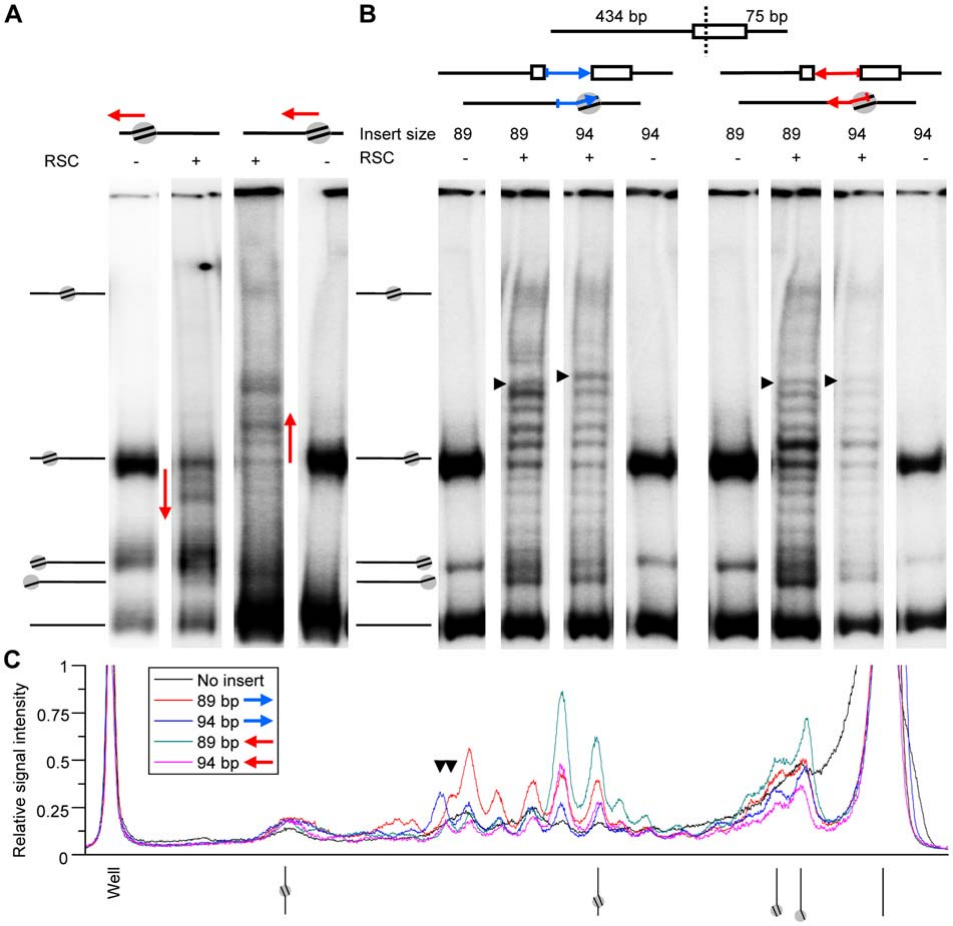
DNA sequence directed initial translocation direction. Native 4% acrylamide gel with or without 5 nM RSC on 11.5 nM mononucleosomes for 1 hour and 100 µM ATP. A) Comparison of initial mononucleosome migration directionality using a 601 nucleosome with reversed left and right arm lengths. The red arrows indicate the preferred direction of initial nucleosome translocation. B) Comparison of mononucleosomes with a 89 or 94 bp insert of pBluescript DNA inserted in either orientation (blue and red arrow) at the *Pml*I site located 50 bp from the middle of the 601 positioning element. The extra 5 deoxyguanosines of the 94 bp insert are positioned at the side of the arrowhead. The black arrowheads indicate the position of the fifth band from the nucleosome starting position. C) Graph of the relative signal intensity of the acrylamide gel lanes containing remodeled nucleosomes with DNA inserts (coloured lines) and without insert with the initial nucleosome translocation direction towards the long DNA arm (black line).

Like previous studies [38], [39], [42]–[44], [51], [59] we used a strong nucleosome positioning sequence to create a homogenous substrate to describe ATP-dependent nucleosome translocation. However, our data show that the positioning sequence itself affects the nucleosomal translocation processivity. Close inspection of the intensity of the nucleosome band pattern in Figure 5 also reveals that both remodelers prefer to slide nucleosomes towards the shortest DNA arm. To determine whether this directional preference is driven by the DNA sequence or the proximity of a DNA end, we inverted the lengths of the flanking arms without changing the orientation of the 601 sequence (Figure 8A). Both remodelers preferred to move the nucleosomes in the same direction with respect to the 601 sequence (Figure 8A and data not shown). This demonstrates that the initial direction of catalyzed nucleosome translocation is determined by the underlying 601 DNA sequence rather than the length of the two flanking DNA arms, ruling out DNA-end proximity effects as the underlying cause of this observation.

### DNA sequence controls nucleosome destination

To learn more about the influence of the 601 sequence on the 10 bp nucleosome remodeling pattern, we inserted 89 or 94 bp DNA fragments in either orientation into the *Pml*I site located 50 bp from the dyad at the side of preferred nucleosome sliding (Figure 8B). The only difference between both inserts is that the 94 bp insert has five extra deoxyguanosines at one end. The inserts did not affect reconstitution specificity as approximately 90% of the nucleosomes were positioned at the original 601 dyad position (Figure 8B). With one exception, the overall step size of the ladder pattern and the nucleosome sliding direction preference were also not changed by the inserts, which reveals the strong influence of the 601 dyad position and its flanking 50 bp on these characteristics of nucleosome repositioning. However, the intensity of the bands in the ladder pattern changed subtly upon insertion of either DNA insert orientations. Furthermore, in the insert orientation with the 5 extra nucleotides positioned 50 bp from the 601 dyad (Figure 8B, blue arrow), the fifth remodeling step was larger for the 94 bp insert than for the 89 bp insert. This difference was not observed for the inserts with the 5 extra nucleotides positioned 139 bp from the 601 dyad (red arrow). These results suggest that DNA sequence variations as small as 5 bp can influence the destination of a catalytically translocated nucleosome.

## Discussion

To date, detailed enzymatic nucleosome mobilization assays have largely employed mobilization over short distances (36–180 bp) from one positioning element to another or towards a nearby end of the DNA molecule [38], [39], [41]–[44]. While these assays demonstrate nucleosome movement away from the initial position as such, they cannot resolve nucleosome translocation processivity and separate the effect of underlying DNA sequences and nearby DNA ends. Studies involving nucleosome remodeling on DNA minicircles were able to show that SWI/SNF moves nucleosomes to more favourable positions on the DNA [40], [60]. However, also with DNA minicircles no conclusions can be drawn concerning the nucleosome translocation processivity and the lack of time courses, enzyme titrations and ATP titrations in these studies made it impossible to draw conclusions on the directionality and sequential steps of catalysed nucleosome repositioning. In this study we have used nucleosomes with DNA extensions of 240–1200 bp together with single molecule AFM and high resolution PAGE. This resulted in an in depth step analysis of ATP-dependent nucleosome repositioning. To assess the generality of our results we compared representative enzymes of two subfamilies of ATP-dependent nucleosome remodeling enzymes including a well known transcription repressor (Mi-2, CHD-type Chromodomain) [25] and an activator (RSC, SWI/SNF-type Bromodomain) [7], [61]. This has allowed us to reveal unexpected properties of SNF2 enzyme-catalyzed nucleosome movement, such as the capacity of DNA sequence to influence nucleosome translocation direction, translocation processivity and final destination.

### Influence of DNA ends on nucleosome translocation

The major advantage of using mononucleosomes with long DNA arms over substrates with DNA arms that protrude only several tens of bp from the nucleosome is that DNA end effects can be separated from DNA sequence induced positioning effects. Though deposition biases cannot be excluded, our AFM imaging suggests that RSC has a higher affinity for DNA ends than for regular DNA and nucleosomes. This affinity for DNA ends may be related to the involvement of RSC in DNA double strand break repair pathways [12], [18], [62] as it is predicted to direct RSC to double strand breaks. This also implies that the RSC complex first binds to and translocates over DNA before engaging in nucleosome remodeling. Nevertheless, we found no evidence that DNA end binding of RSC influences the initial direction of nucleosome translocation. It is therefore likely that under our experimental conditions translocation of the nucleosome itself rather than the binding to and translocation of RSC over bare DNA was rate limiting. A clear separation between RSC translocation on bare DNA and of a nucleosome may be the key to resolve the discrepancy between recent single-molecule experiments concerning the translocation velocity on DNA and nucleosomal DNA [37], [54], [63], [64].

Another DNA end effect apparent from both our AFM and PAGE analyses was that histone octamers were pushed some tens of bp over the end by RSC, a finding that is consistent with previous work on RSC and SWI/SNF [38], [43], [52], [53]. This was not the case when Mi-2 was used. The accumulation of mobilized nucleosomes at DNA ends suggests that RSC and SWI/SNF cannot efficiently reposition nucleosomes from there, indicating the requirement of bare DNA upstream of the nucleosome translocation direction and consistent with a reaction mechanism in which binding to bare DNA would precede nucleosome repositioning. Finally, a mechanism whereby nucleosomes are ‘pushed’ rather than ‘pulled’ may also explain how RSC and SWI/SNF force adjacent nucleosomes into overlapping positions [51], [65].

### DNA sequence-directed nucleosome translocation

The effect of the DNA sequence on ATP dependent nucleosome translocation processivity was clearly demonstrated by the strong artificial 601 nucleosome phasing sequence, which severely constrained the translocation processivity of both remodelers [45]. Using Mi-2, few nucleosomes ‘escaped’ the 601 sequence, whereas with RSC, the processivity was several fold lower within its influence than beyond. This indicates that both DNA sequence and intrinsic enzyme properties must be taken into account to describe the mechanism of catalyzed nucleosome translocation. This seemingly contrasts with Partensky et al. [41] who argue that yeast RSC and human ISWI containing ACF complexes are as efficient in repositioning nucleosomes away from the initial position on DNAs with widely divergent octamer affinities and nucleosome breathing dynamics. However, their results could also be interpreted as unwrapping of DNA from the histone octamer, rather than nucleosome repositioning. The rather short DNA fragments used in their study with the initial nucleosome positioned at the DNA end may have influenced the results, as we show here that DNA ends affect RSC remodeling.

Another prominent 601 sequence-related feature that we observed for both remodelers was a 10 bp periodicity of the reaction products. Schwanbeck et al. [44] also observed two 10 bp steps of the ISWI-type NURF complex on the 601 sequence with 47 bp linker DNA and they report these steps as remodeler specific. This synthetic DNA sequence displays a 10 bp periodicity of the dinucleotides TA, TT and AA together with an out of phase 10 bp periodicity of the dinucleotide GC [66] resulting in a free energy which is approximately 3 k_b_T less than the naturally occurring 5S rDNA or MMTV nucleosome phasing sequences [45], [67], [68]. Though the nucleosome affinity of natural positioning sequences is less strong than the artificial 601 sequence, they also display a 10 bp periodicity [66], [69], [70]. We therefore argue that natural positioning sequences may influence SNF2 enzyme reactions in a similar way as the 601 phasing sequence does.

We revealed that the newly adopted nucleosome positions after enzymatic remodeling are influenced by the underlying DNA sequence by inserting DNA fragments into the 601 positioning element. Partensky et al. [41] obtained a similar result when inserting a DNA fragment just next to the 601 sequence. These changes in are likely due to the local changes in the thermodynamic landscape of the DNA sequence [38], [71].

Shundrovsky et al. [72] suggested that catalyzed sliding of nucleosomes from the 601 position is direction independent. However, a striking DNA sequence-dependent effect we report here is that the 601 mononucleosome is asymmetric in that both remodelers displayed pronounced preference for translocation of the nucleosome into the direction of the *Pml*I site of the 601 sequence, independent of the lengths of the flanking DNA arms. Such DNA sequence mediated nucleosome anisotropy may be important for specific nucleosomes located strategically within chromosomes, such as those flanking nucleosome free regions since it would contribute information as to the direction that a nucleosome will preferentially slide towards *in vivo*, which may have far-reaching regulatory consequences.

### SNF2-dependent nucleosome translocation

Having separated the effects of DNA sequence and DNA ends on catalyzed nucleosome repositioning, the question remains as to what distinguishes the reaction mechanisms of different remodelers with specialized functions in chromatin maintenance. The first obvious difference was that native RSC proved to be a more processive enzyme than recombinant Mi-2. Whereas the kinetics of Mi-2 within the 601 sequence was consistent with a repositioning of 10 bp per turnover, RSC displayed a processivity consistent with approximately 20 bp within the 601 sequence, which increased to over 80 bp per turnover beyond the influence of the 601 sequence. This difference could be due to differences in enzyme complexity. Indeed, it has been shown that recombinant Sth1p, the ATPase of RSC, is less active than native RSC complex [73]. When in a protein complex, subunits with DNA or histone binding sites are likely to increase processivity by increasing the translocation velocity or lowering dissociation constants, as exemplified by the large DNA footprint and the putative encapsulation of the nucleosome in the central cavity of the RSC complex.

Unlike Mi-2, RSC displayed a preferred nucleosomal step size of two times 10 bp within the 601 sequence under limiting ATP conditions. This result suggests that RSC has a default nucleosomal translocation step size between 15 and 25 bp. The fact that limiting ATP concentration affected processivity rather than step-size is consistent with a ‘DNA pumping’ or ‘loop diffusion’ model, but does not exclude the involvement of twist [33], [34], [37], and could be taken to suggest that a single DNA translocation event may be performed upon as few as one ATPase cycle, following a swivelling motion of a part of the ATPase, similar to what has been proposed for SFII superfamily DNA helicases [74].

### Conclusions

Aside from the differences between the remodelers tested, we show here that the DNA sequence is a key factor in defining the extent and direction of ATP-dependent nucleosome translocation. DNA sequence therefore controls catalyzed nucleosome repositioning *in vitro*. Strongly positioned nucleosomes may depend on ATP-dependent chromatin remodelers to be displaced *in vivo*, perhaps with the assistance of sequence specific DNA binding factors [75], [76] and/or histone chaperones [77]. This would free up nucleosomal DNA to enable the initiation of processes such as transcription, replication, repair or recombination. A high nucleosome translocation processivity beyond the influence of strong positioning elements, such as that displayed here by RSC outside the 601 sequence, may help processive DNA metabolic processes, including elongation by RNA polymerase [7] and perhaps even migration through chromatin of replication forks or Holliday junctions.

Overall, the present combination of biochemical and biophysical analyses shows that SNF2-type ATP-dependent nucleosome remodelers allow control of their reactions by DNA sequence. This may explain the apparent paradox between the need for a high abundance of ATP-dependent remodelers per nucleus, ∼1 per 12 nucleosomes in yeast [31], [78], [79], and the high success rate of sequence based predictions achieved for nucleosome positioning *in vivo* [70]. Here we propose a three-step framework to describe SNF2 enzyme mediated nucleosome sliding that consists of an initiation step that defines the direction of migration, followed by one or more translocation steps whose length and number depend on the particular SNF2 enzyme recruited combined to the properties of the underlying DNA sequence, and a third step that releases the nucleosome at DNA sequences that are energetically favourable.

## Materials and Methods

### Remodeler isolation

Native RSC complex was isolated from a yeast strain via C-terminal tagged Npl6p using the Tandem Affinity Purification protocol essentially as described by Puig et al. [80] and further modified by Campsteijn et al. [14], 400 mM KAc was used instead of 150 mM NaCl. The integrity of the RSC complex was confirmed by silver staining the complex on a denaturing polyacrylamide gel (Figure S5). Recombinant *Drosophila* Mi-2 was isolated as described previously [42].

### DNA and nucleosomal substrate

DNA fragments were prepared by PCR or restriction enzyme digestion using the pGEM-3Z vector containing a single 601 positioning element (kind gift of J. Widom). Mononucleosomes were generated by salt dialysis [81]. The 1 kbp bare DNA fragment without nucleosomal positioning element was obtained from Eurogentec. The centrally positioned nucleosomes in our AFM experiments were generated with 635 bp and 2550 bp long DNA (PCR) and recombinant *Xenopus leavis* octamers [82]. The nucleosomes used in our PAGE experiments were generated with purified *Gallus gallus* erythrocyte histone octamers [81], 656 bp long DNA for centrally positioned nucleosomes (601 pGEM-3Z, *Pvu*II digestion) and 660 bp DNA for off-centre nucleosomes (PCR). Primer sequences are available on request.

### AFM

Remodeling reactions were performed in 100 mM NaAc, 10 mM MgAc, 10 mM Hepes pH 8 and 4 mM ATP for 30 min at room temperature. To image RSC-DNA complexes the remodeling reaction was 10 times diluted with deposition buffer (10 mM Hepes-KOH pH 8, 10 mM MgCl_2_) of which 5 µl was deposited on freshly cleaved mica. To image RSC-mono-nucleosome complexes the remodeling reaction was fixed with 0.1% glutaraldehyde for 15 minutes at room temperature. Next, 5 µl of the reaction was deposited on a poly-l-lysine coated mica surface. Before deposition the 1200/1200 bp nucleosomes were five times diluted with deposition buffer. Deposited samples were flushed with ultrapure water, dried with nitrogen gas and imaged using a Nanoscope IV (Digital Instruments) operated in tapping mode AFM.

### Determination of DNA contour length and position of protein complexes

The contour length of a DNA molecule was determined by tracing the DNA from end to end. Internally bound RSC complexes were traced by linking the DNA entry and exit points in a straight line through the complex. The position of the RSC complex was then determined in the centre of the straight line. Internally bound nucleosomes were traced with a straight line from the DNA entry point to the nucleosome centre, followed by a straight line from the nucleosome centre to the DNA exit point. For end positioned RSC complexes and nucleosomes the trace follows a straight line from the DNA entry point through the centre of the protein complex to the complex end. A Gaussian fit was applied to all histograms of DNA contour lengths. The error in all other histograms was calculated using the square root of the number of molecules.

### Native acrylamide gel electrophoresis

Remodeling reactions were performed in 50 mM KAc, 2.5 mM MgAc, 2 mM Hepes pH 7.8, 100 ng/ml BSA and 1 mM DTT at room temperature. The reactions were stopped by adding 1 µg native chicken erythrocyte oligonucleosomes per reaction. Glycerol was added to a final concentration of 9% and the samples were loaded on a 4% acrylamide gel (19∶1 acrylamide∶bisacrylamide in 1x TBE buffer) and run at 4°C at 50 V for 8-16 hours.

### Monte Carlo modelling of nucleosome translocation

The Markov chain model of nucleosome translocation shown in Figure 7A was simulated in a Monte Carlo program written in LabView. For each simulation the substrate length and nucleosome position were set according to the experimental setup. From a pool of 10^4^ nucleosomes in successive iterations a single nucleosome was selected randomly and shifted over the DNA in either direction with equal chance. The nucleosome translocation distance depends on the step size and processivity and is defined by an exponential distribution. Accordingly, for each turnover a translocation distance was generated following Poisson statistics. In the case of RSC remodeling all nucleosomes that reached the DNA end were trapped. At exponentially increasing iteration intervals the position distribution of the entire pool of nucleosome positions was calculated. To allow for comparison with PAGE experiments the nucleosome position distribution was folded around the central position, plotted on an exponential scale and convoluted with a point spread function representing the band shape observed in the PAGE experiments. With a constant step size of 10 bp, the processivity and average number of turnovers per molecule were adjusted to match the experimental outcome.

## Supporting Information

Figure S1 Bare DNA with end-bound RSC. The left panel shows the histogram of the contour length of 1 kbp DNA with RSC bound to its end (*N* = 48). The right panel shows an AFM image of a DNA molecule with RSC bound to its end. Scale bar is 100 nm, z-range is 4 nm. White arrows indicate the 2 small lobes of RSC.
(0.87 MB TIF)

Figure S2 Nucleosomes at the DNA end. The top panel shows the histogram of the DNA contour length of internally positioned nucleosomes (category 1, *N* = 49). The middle panel shows the histogram from the contour length of nucleosomes from the same remodeling reaction that were repositioned to the DNA end (category 7, *N* = 62). The bottom panel shows an AFM image of nucleosomes with 240 bp arms, one internally bound and one bound to the DNA end. Scale bar is 100 nm, z-range 4 nm.
(1.11 MB TIF)

Figure S3 Mi-2 time course on off-centre nucleosomes, with simulation. Native 4% acrylamide gel of a time course of 5 nM Mi-2 on 11.5 nM mononucleosomes with a 75 and 434 bp arm and 1 mM ATP and Monte-Carlo simulation assuming Mi-2 initially binds at the nucleosome, translocates it with a 10 bp step size and reflects the nucleosome from the DNA end (upper panel). The graphs in the lower panel show the relative signal intensity of the lanes of the acrylamide gel and Monte-Carlo simulation in the upper panel, whereby a relative signal intensity of 1 corresponds to the highest peak in the graph.
(4.60 MB TIF)

Figure S4 Simulation of centrally positioned nucleosome remodeling by RSC. Native 4% acrylamide gel with 0–10 nM RSC titration on 7.7 nM nucleosomes with a 204 and a 309 bp arm for 1 hour with 1 mM ATP (reappearance of Figure 4A) and Monte-Carlo simulation assuming RSC binds at the nucleosome, translocates with a 10 bp step size, 80 bp processivity and is not able to reposition the nucleosome from the DNA end (upper panel). The graphs in the lower panel show the relative signal intensity of the lanes of the acrylamide gel and Monte-Carlo simulation in the upper panel, whereby a relative signal intensity of 1 corresponds to the highest peak in the graph.
(2.61 MB TIF)

Figure S5 Silver stained native RSC complex. Silver staining of 10% polyacrylamide gel with 0.1% SDS on which 0.1 pmol tandem affinity purified RSC was run.
(0.83 MB TIF)
