# Supplementary figures and images for: Multiple Aspects of ATP-Dependent Nucleosome Translocation by RSC and Mi-2 Are Directed by the Underlying DNA Sequence
Source: PLoS One. 2009 Jul 23;4(7):e6345. doi: 10.1371/journal.pone.0006345 (PMC2710519; doi:10.1371/journal.pone.0006345)

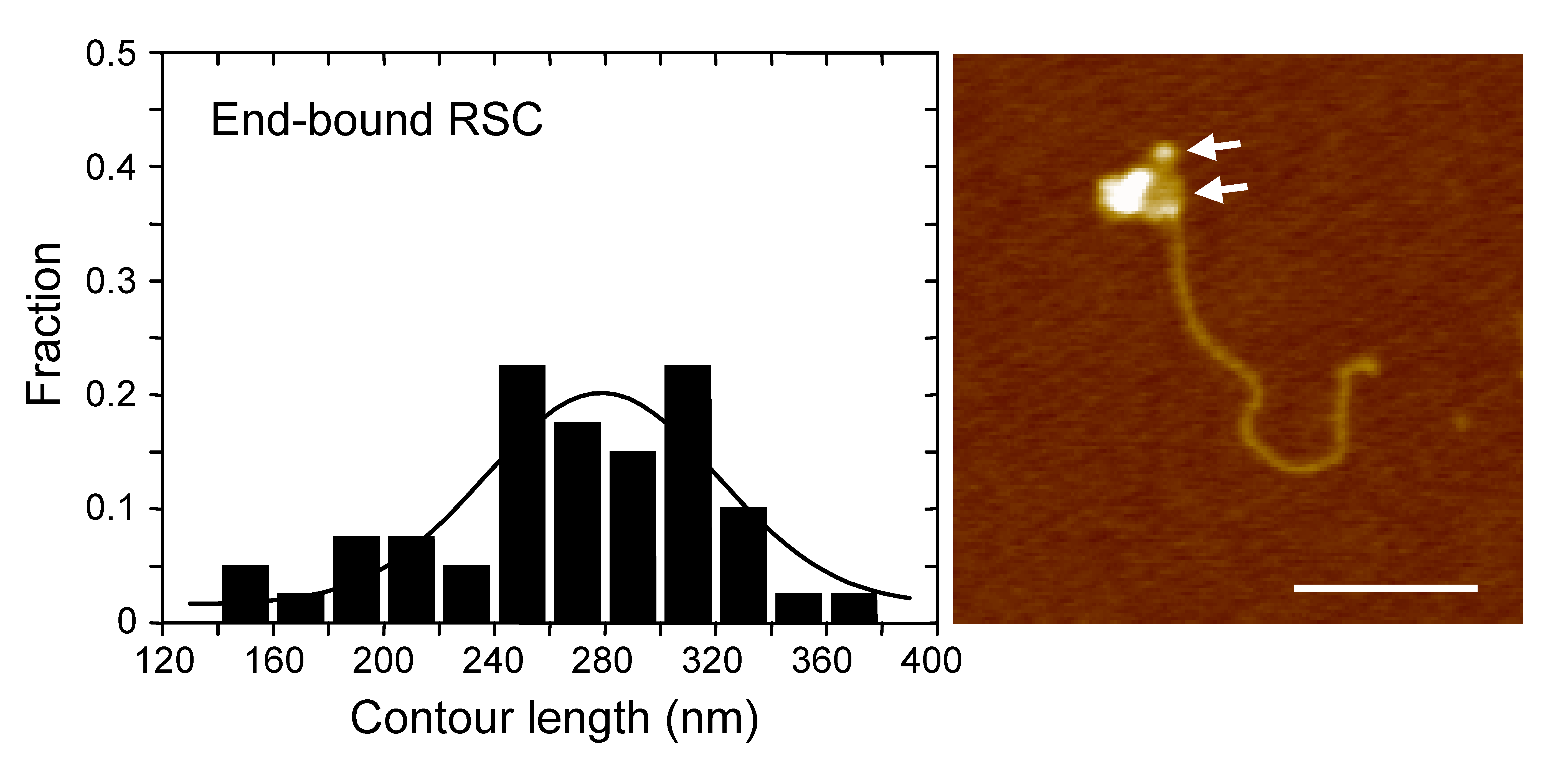

Supplement: Figure S1 — Bare DNA with end-bound RSC. The left panel shows the histogram of the contour length of 1 kbp DNA with RSC bound to its end (N = 48). The right panel shows an AFM image of a DNA molecule with RSC bound to its end. Scale bar is 100 nm, z-range is 4 nm. White arrows indicate the 2 small lobes of RSC. (0.87 MB TIF) [file pone.0006345.s001.tif]

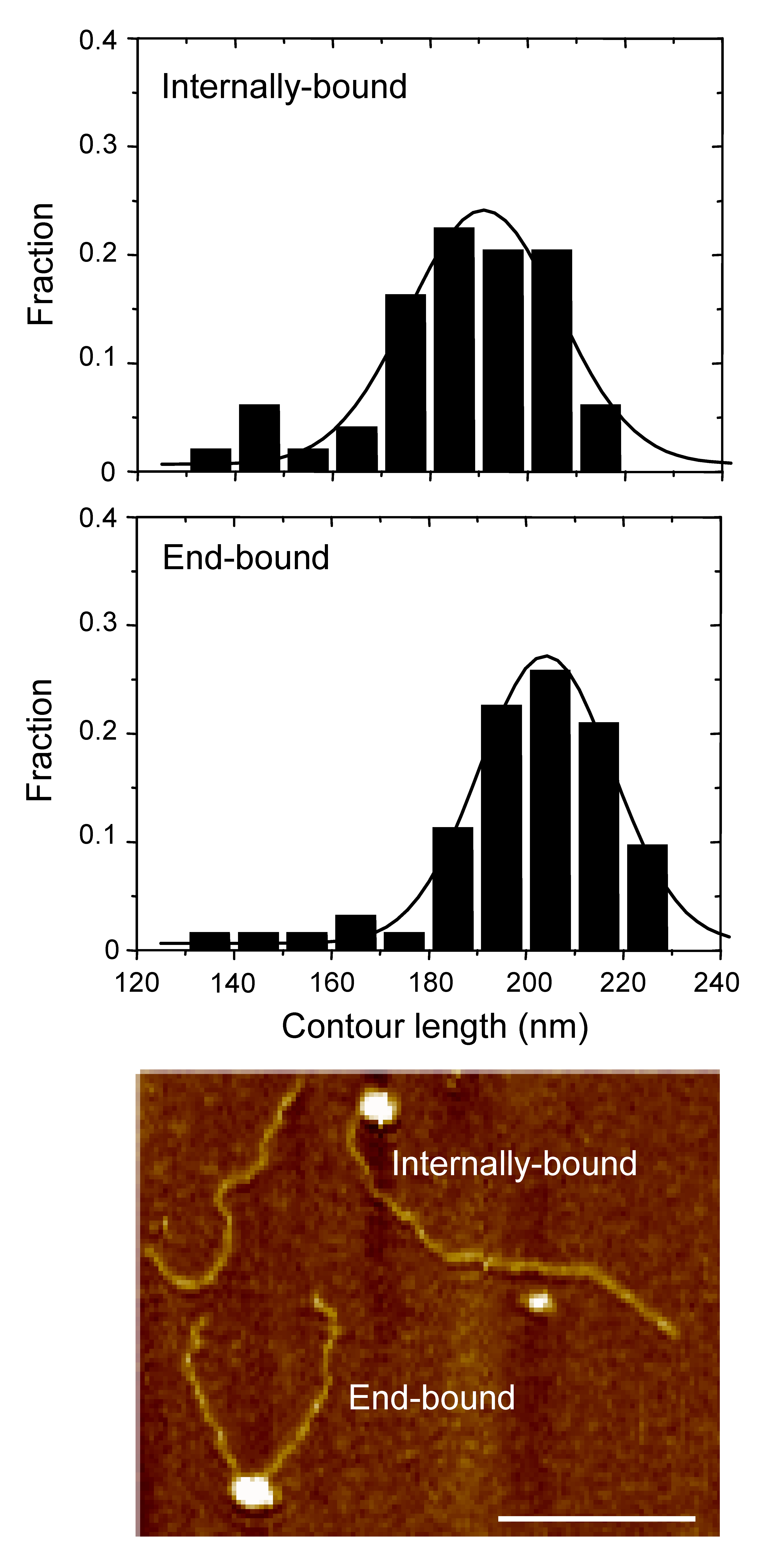

Supplement: Figure S2 — Nucleosomes at the DNA end. The top panel shows the histogram of the DNA contour length of internally positioned nucleosomes (category 1, N = 49). The middle panel shows the histogram from the contour length of nucleosomes from the same remodeling reaction that were repositioned to the DNA end (category 7, N = 62). The bottom panel shows an AFM image of nucleosomes with 240 bp arms, one internally bound and one bound to the DNA end. Scale bar is 100 nm, z-range 4 nm. (1.11 MB TIF) [file pone.0006345.s002.tif]

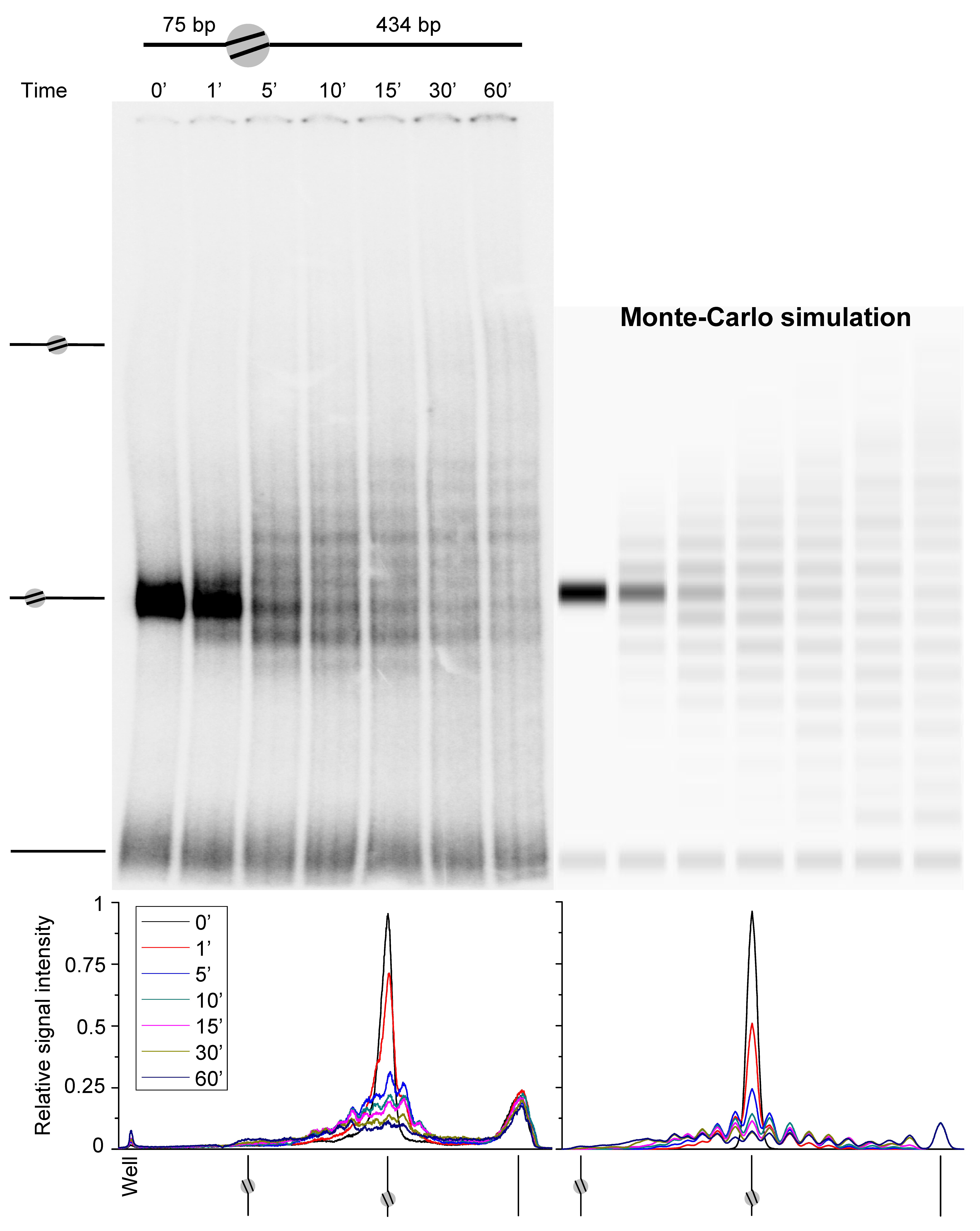

Supplement: Figure S3 — Mi-2 time course on off-centre nucleosomes, with simulation. Native 4% acrylamide gel of a time course of 5 nM Mi-2 on 11.5 nM mononucleosomes with a 75 and 434 bp arm and 1 mM ATP and Monte-Carlo simulation assuming Mi-2 initially binds at the nucleosome, translocates it with a 10 bp step size and reflects the nucleosome from the DNA end (upper panel). The graphs in the lower panel show the relative signal intensity of the lanes of the acrylamide gel and Monte-Carlo simulation in the upper panel, whereby a relative signal intensity of 1 corresponds to the highest peak in the graph. (4.60 MB TIF) [file pone.0006345.s003.tif]

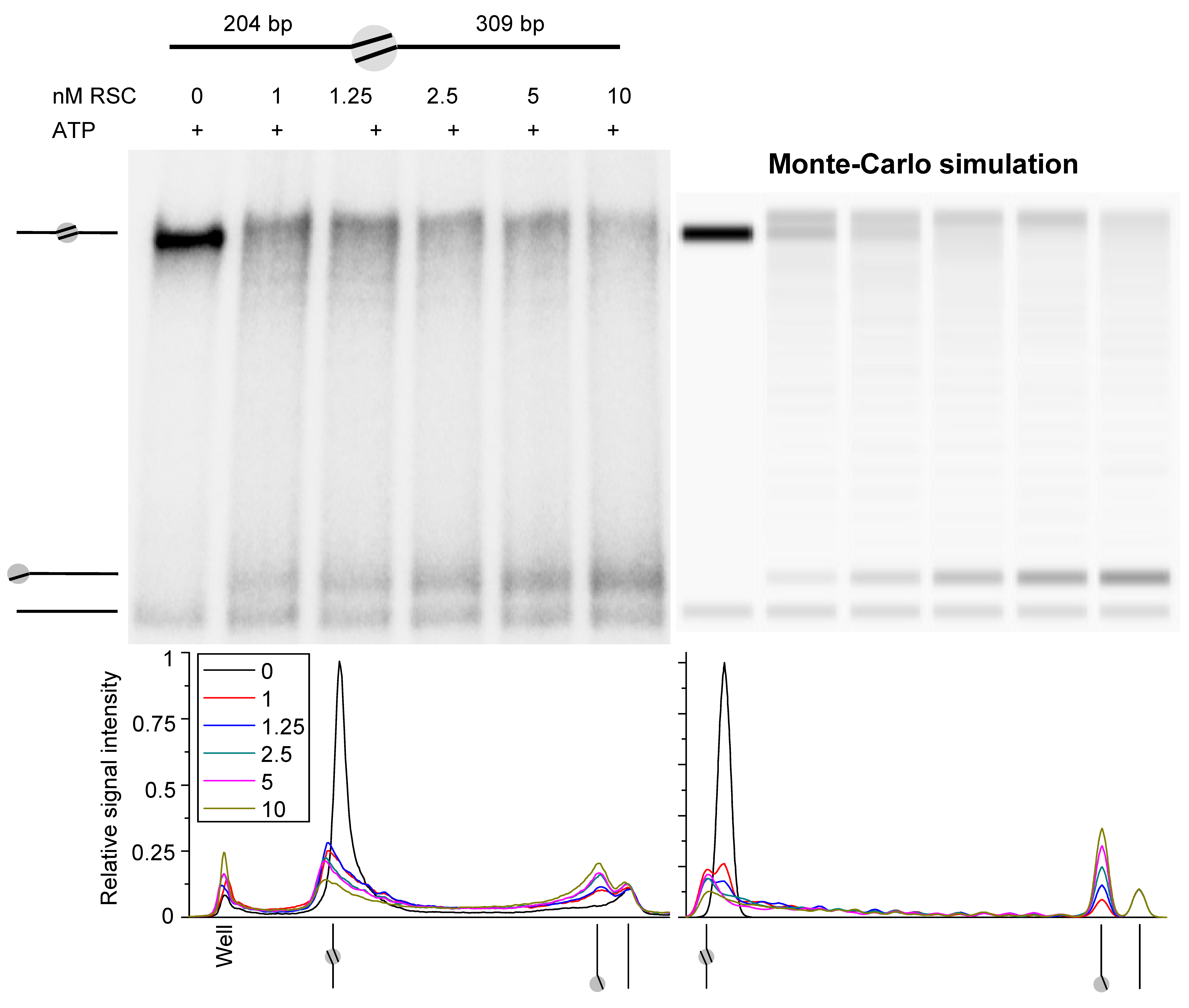

Supplement: Figure S4 — Simulation of centrally positioned nucleosome remodeling by RSC. Native 4% acrylamide gel with 0–10 nM RSC titration on 7.7 nM nucleosomes with a 204 and a 309 bp arm for 1 hour with 1 mM ATP (reappearance of Figure 4A) and Monte-Carlo simulation assuming RSC binds at the nucleosome, translocates with a 10 bp step size, 80 bp processivity and is not able to reposition the nucleosome from the DNA end (upper panel). The graphs in the lower panel show the relative signal intensity of the lanes of the acrylamide gel and Monte-Carlo simulation in the upper panel, whereby a relative signal intensity of 1 corresponds to the highest peak in the graph. (2.61 MB TIF) [file pone.0006345.s004.tif]

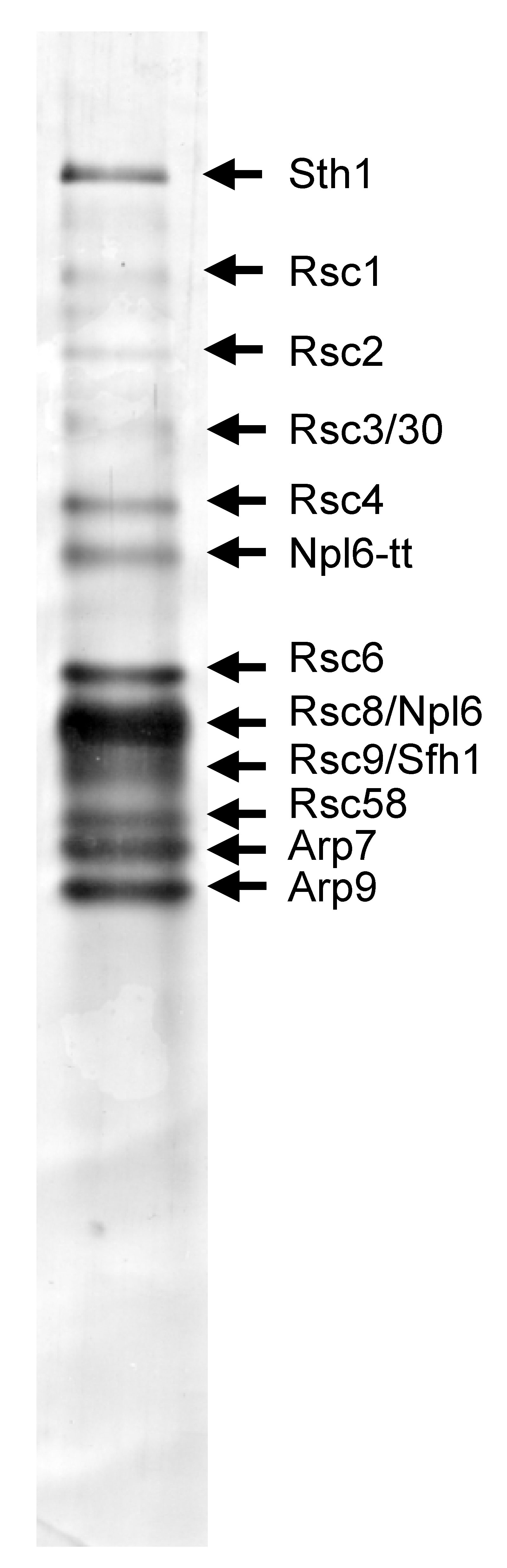

Supplement: Figure S5 — Silver stained native RSC complex. Silver staining of 10% polyacrylamide gel with 0.1% SDS on which 0.1 pmol tandem affinity purified RSC was run. (0.83 MB TIF) [file pone.0006345.s005.tif]
